# Assessment of the Glycan-Binding Profile of Pseudomonas aeruginosa PAO1

**DOI:** 10.1128/spectrum.01667-23

**Published:** 2023-07-20

**Authors:** Hector Sanchez, George A. O’Toole, Brent Berwin

**Affiliations:** a Department of Microbiology and Immunology, Geisel School of Medicine at Dartmouth, Lebanon, New Hampshire, USA; b The Jackson Laboratory, Bar Harbor, Maine, USA; Emory University School of Medicine

**Keywords:** *Pseudomonas aeruginosa*, glycan, array, phagocytosis, host-pathogen interactions

## Abstract

Pseudomonas aeruginosa is an opportunistic pathogen that can establish acute and chronic infections in individuals who lack fully functional innate immunity. In particular, phagocytosis by neutrophils and macrophages is a key mechanism that modulates host control and clearance of P. aeruginosa. Individuals with neutropenia or cystic fibrosis are highly susceptible to P. aeruginosa infection, thus underscoring the importance of the host innate immune response. Cell-to-cell contact between host innate immune cells and the pathogen, a first step in phagocytic uptake, is facilitated by simple and complex glycan structures present at the host cell surface. We have previously shown that endogenous polyanionic N-linked glycans localized to the cell surface of phagocytes mediate the binding and subsequent phagocytosis of P. aeruginosa cells. However, the suite of glycans that P. aeruginosa cells bind to on host phagocytic cells remains poorly characterized. Here, we demonstrate, with the use of exogenous N-linked glycans and a glycan array, that P. aeruginosa PAO1 cells preferentially attach to a subset of glycans, including a bias toward monosaccharide versus more complex glycan structures. Consistent with these findings, we were able to competitively inhibit bacterial adherence and uptake by the addition of exogenous N-linked mono- and disaccharide glycans. We discuss our findings in the context of previous reports of P. aeruginosa glycan binding.

**IMPORTANCE** P. aeruginosa cells bind to a variety of glycans as part of their interaction with host cells, and a number of P. aeruginosa-encoded receptors and target ligands have been described that allow this microbe to bind to such glycans. Here, we extend this work by studying the glycans used by P. aeruginosa PAO1 cells to bind to phagocytic cells and by using a glycan array to characterize the suite of such molecules that can facilitate host cell binding by this microbe. This study provides an increased understanding of the glycans bound by P. aeruginosa and furthermore provides a useful data set for future studies of P. aeruginosa-glycan interactions.

## INTRODUCTION

Pseudomonas aeruginosa is a Gram-negative, opportunistic bacterium that is responsible for a variety of human infections, particularly in individuals from immunocompromised communities. P. aeruginosa is capable of losing flagellar swimming motility during chronic infection, likely secondary to forming a biofilm, which in turn allows this microbe to evade immune responses via phagocytic resistance and antibiotic tolerance (1–5). Persons with cystic fibrosis (CF) and neutropenia (lacking neutrophils) are highly susceptible to bacterial infections due to defects in phagocytic function preventing clearance of P. aeruginosa (6, 7).

Phagocytosis by neutrophils and macrophages provides the host with an effective defense mechanism against bacterial infection (8). Contact between the host immune cell and the microbe is required for initiation of phagocytosis. We previously identified that exogenous treatment of phagocytes with a negatively charged phosphoinositide, PIP_3_, promotes phagocytosis of nonmotile P. aeruginosa cells by increasing binding to phagocytes (9). These studies, which suggested that P. aeruginosa cells bind to clustered polyanions, guided our previous work, which revealed that endogenous negatively charged glycosaminoglycans (GAGs) can mediate P. aeruginosa adhesion. In turn, this observation led to the conclusion that N-linked glycans on phagocytes play a role in bacterial binding and phagocytosis (10). Therefore, a central goal of this study was to further elucidate the initial carbohydrate “handshake” between phagocytic cells and P. aeruginosa.

Glycans are found in all of nature and cover cell surfaces by decorating protein and lipid backbones (11, 12). Glycans on host cells are often utilized by pathogenic bacteria for attachment and invasion (13–16) and can even serve as a carbon/energy source (17). The binding of P. aeruginosa cells to glycans has been explored previously in several contexts. We showed that N-linked glycans and glycosaminoglycans on phagocytes can mediate the attachment and uptake of P. aeruginosa by macrophages (10). N-linked glycans have also been implicated as ligands for bacterial attachment to epithelial cells (18, 19). P. aeruginosa cells can also bind glycan components of mucin (20–23), complex glycans as part of glycolipids (24, 25), and chitin (26, 27).

In this work, we further explore the features of N-linked glycan structures that promote P. aeruginosa PAO1 binding, including mannose (Man), glucose (Glc), *N*-acetylglucosamine (GlcNAc), galactose (Gal), and fucose. To do so, we utilized single monosaccharide components of an N-linked glycan structure in competition assays. In parallel, we also performed a glycan array study to further evaluate the binding profile of P. aeruginosa to different classes of glycans. These studies have led to a better understanding of the glycans that can be bound by P. aeruginosa PAO1 and furthermore provides a useful data set for others investigating P. aeruginosa-glycan interactions.

## RESULTS

### Exogenous monosaccharides compete the phagocytosis of P. aeruginosa by THP-1 cells.

We previously demonstrated that if N-linked glycan synthesis is inhibited on host phagocytic cells, the ability of P. aeruginosa cells to bind to the cell surface via these glycans is reduced (10). We hypothesized that the sugars that comprise N-linked glycan structures are necessary for the binding and uptake of P. aeruginosa. These N-linked glycan structures are primarily composed of mannose, *N*-acetylglucosamine, fucose, galactose, and sialic acid moieties, depending on the cell type; furthermore, N-linked glycans can be present in different quantities (28). There are three different classes of N-linked glycans (high mannose, complex, and hybrid), and each of these glycans share a core structure of Man_3_GlcNAc_2_ bound to an asparagine (Asn) residue (29).

To assess the binding of glycan structures by P. aeruginosa cells, we performed competition assays in which we exogenously supplemented phagocytosis assays with components of N-linked glycans. In this set of experiments, we utilized P. aeruginosa PAO1, a motile strain of bacteria previously shown to interact with cell surface glycans (10, 30), and performed gentamicin protection assays with human THP-1 monocytic cell lines, as reported (4, 5, 9, 10), to assess the interactions of the microbe with host cells. The assays were performed for 45 min, which we showed previously allows for a reproducible measure of bacterial attachment and subsequent phagocytosis, with minimal change in the viability of the internalized bacteria (4, 5, 9, 10).

**FIG 1:**
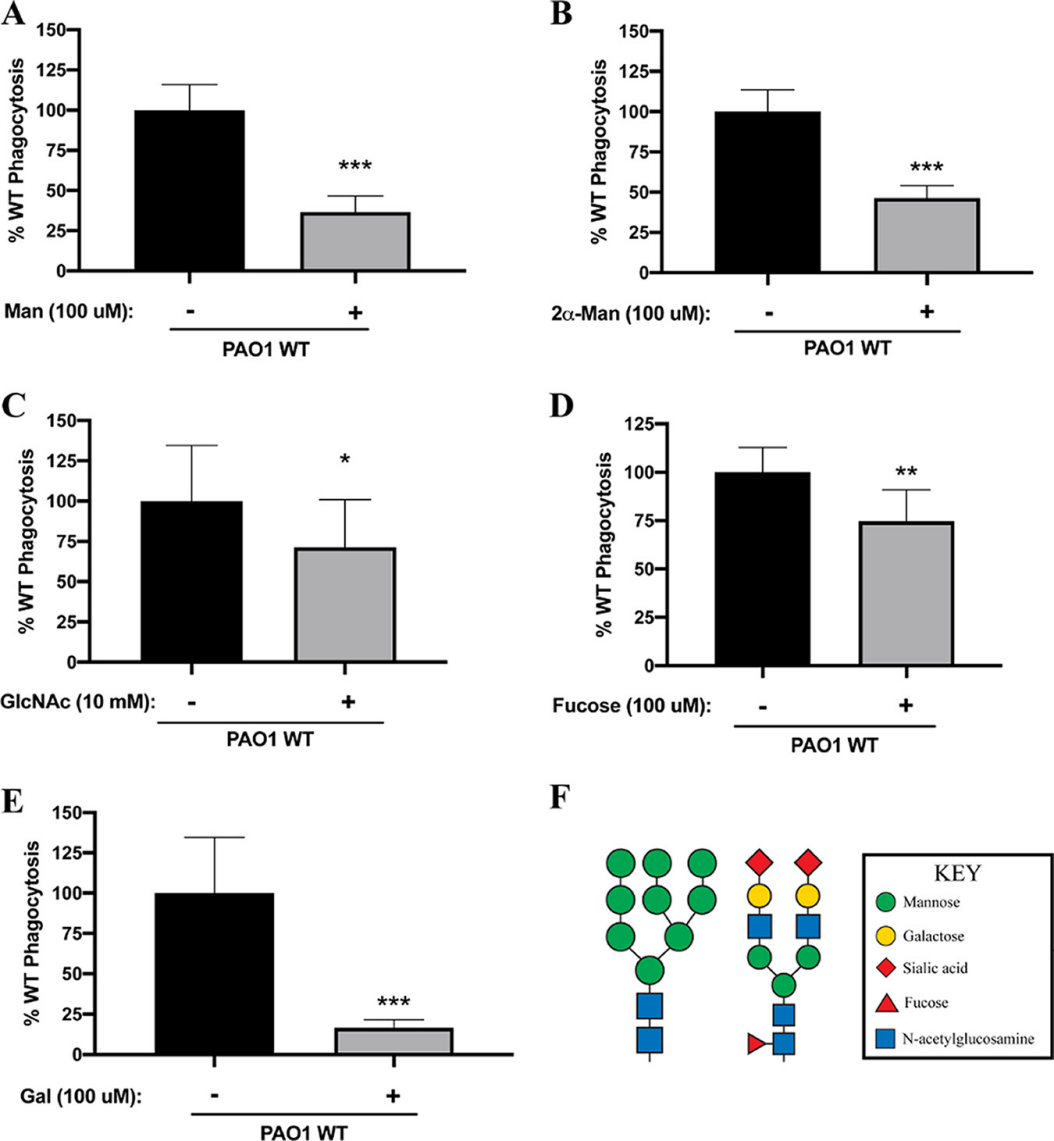
Competition with mono- and disaccharide components of N-linked glycans inhibits P. aeruginosa PAO1 phagocytosis. THP-1 cells were assayed for relative phagocytosis of P. aeruginosa PAO1 (MOI = 10) in the absence or presence of the indicated exogenously added sugars. Phagocytosis was normalized as a percentage of the mean of phagocytosis of P. aeruginosa cells by untreated THP-1 cells. (A to E) Mannose (Man; 100 μM) (A), 2α-mannobiose (2α-Man; 100 μM) (B), *N*-acetylglucosamine (GlcNAc; 10 mM) (C), fucose (100 μM) (D), and galactose (Gal; 100 μM) (E) were assessed in the competition assays. Data in panels A to E were analyzed using an unpaired *t* test with Welch’s correction and are representative of at least three independent biological experiments with at least two technical replicates (*n* ≥ 3). ***, *P ≤ *0.0005; **, *P ≤ *0.005; *, *P* ≤ 0.05. (F) Schematic representation of N-linked structures: high-mannose type (left) and complex type (right). The structures were modified with permission from the figures at https://www.raybiotech.com/.

**FIG 2:**
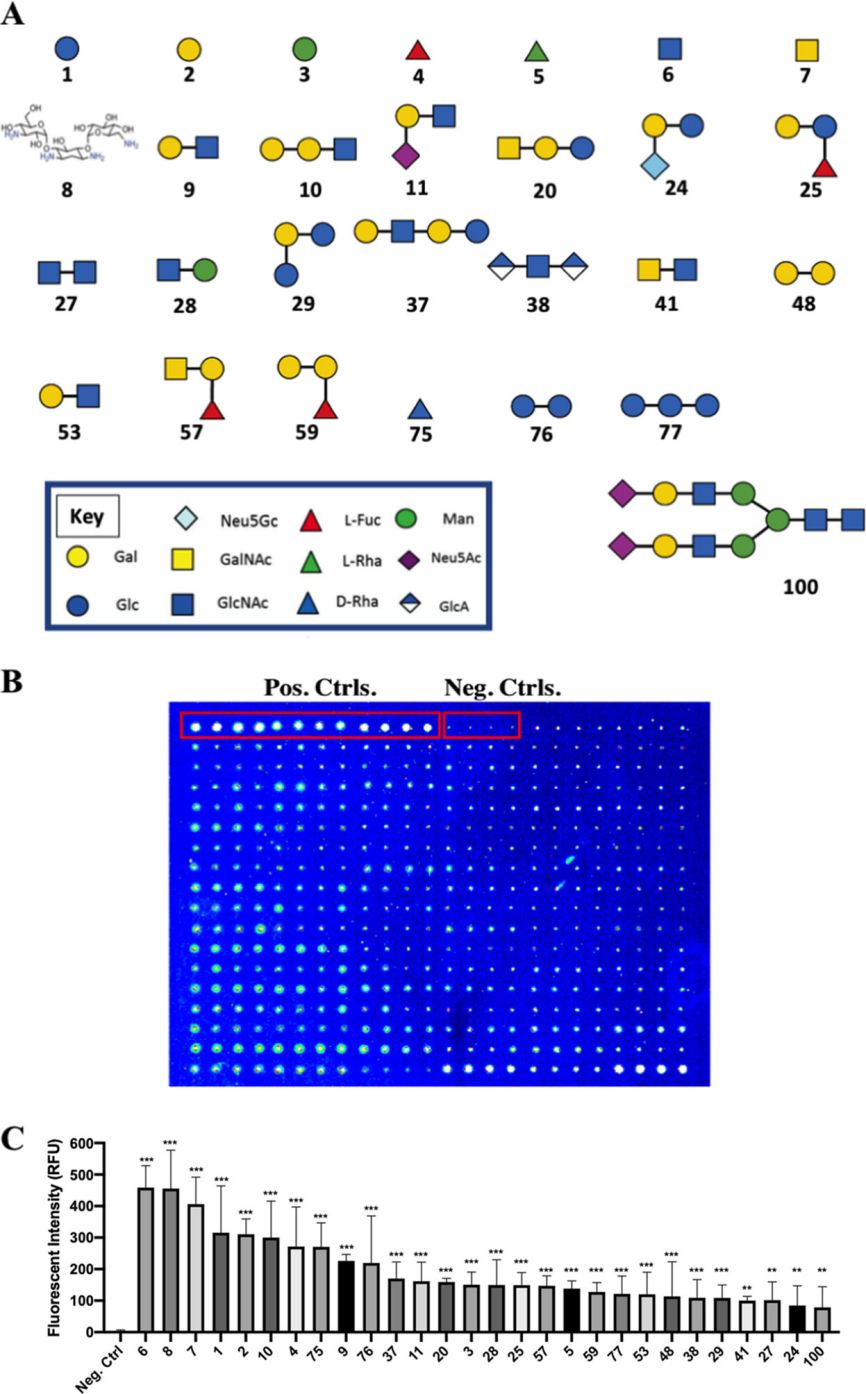
Analysis of glycan binding by P. aeruginosa PAO1 using the Glycan-100 array. GFP-expressing P. aeruginosa PAO1 was quantitatively assessed for binding to known glycan structures printed on the Glycan-100 array. (A) Structures of the 28 compounds significantly bound by P. aeruginosa PAO1. The numbering of the compounds corresponds to that used in the text and the Glycan-100 array. The structures were modified with permission from the figures at https://www.raybiotech.com/. (B) Representative example of one of the arrays tested for glycan binding by P. aeruginosa. The positive control (a fluorescent protein) and negative control (nothing spotted) are labeled and indicated by the red boxes. (C) Glycan array analysis revealed that P. aeruginosa PAO1 cells significantly bound 28 distinct glycans in this assay. The average fluorescence intensity associated with each glycan was compared to that of the negative control. The number on the *x* axis corresponds to the numbering in the text and in panel A of this figure. Data were analyzed using one-way analysis of variance (ANOVA) with Dunnett’s *post hoc* analysis and are derived from three independent experiments with quadruple spotting of each glycan on each array (*n* = 12). ***, *P ≤ *0.0005; **, *P ≤ *0.005.

We first assessed the phagocytosis of P. aeruginosa PAO1 cells by THP-1 cells in the presence of mannose (Fig. 1A) and 2α-mannobiose (Fig. 1B), a disaccharide of mannose linked by a 1-2 glycosidic bond (31). The addition of either of these compounds resulted in significantly reduced (~50%) uptake of P. aeruginosa. The addition of *N*-acetylglucosamine at a relatively high concentration (Fig. 1C) and fucose (Fig. 1D) caused a statistically significant but modest (~20%) reduction in P. aeruginosa phagocytosis. Finally, consistent with our previous observations (10), the internalization of P. aeruginosa was robustly and significantly diminished when the assay was supplemented with free galactose (Fig. 1E). Together, these data are consistent with the conclusion that P. aeruginosa PAO1 cells can bind to various mono- and disaccharide components that comprise high-mannose and complex glycans (Fig. 1F).

### An assay to quantitatively profile interactions between P. aeruginosa and glycans.

To identify additional candidate glycan ligands for P. aeruginosa, we utilized an otherwise wild-type (WT), green fluorescent protein (GFP)-expressing strain of P. aeruginosa PAO1 and performed a bacterial binding assay with the commercially available Glycan-100 array (RayBiotech). Each of the 100 glycan types was spotted in quadruplicate. An overnight culture of P. aeruginosa PAO1 carrying a GFP-expressing plasmid grown in lysogeny broth (LB) was subcultured and grown to mid-log phase in LB and then diluted 1:1,000 to ~1 × 10^6^ cells/mL in the manufacturer’s minimal sample diluent buffer. After a 3-h incubation at room temperature with this bacterial suspension, each array was washed to remove nonbinding cells, and the fluorescence intensity values associated with the spotted glycans were quantified. These experiments were performed in triplicate; additional details of the experiment and its analysis are presented in Materials and Methods.

We observed a total of 28 glycans to which P. aeruginosa cells consistently and significantly bound (Fig. 2A). These glycans are as follows: β-Glc (glycan 1), β-Gal (glycan 2), α-Man (glycan 3), α-Fuc (glycan 4), α-Rha (glycan 5), β-GlcNAc (glycan 6), β-GalNAc (glycan 7), tobramycin (glycan 8), Gal-β-1,3-GlcNAc-β (glycan 9), Gal-α-1,3-Gal-β-1,3-GlcNAc-β (glycan 10), Neu5Ac-α-2,3-Gal-β-1,3-GlcNAc-β (glycan 11), GalNAc-β-1,3-Gal-β-1,4-Glc-β (glycan 20), Neu5Gc-α-2,6-Gal-β-1,4-Glc-β (glycan 24), Gal-β-1,4-(Fuc-α-1,3)-Glc-β (glycan 25), GlcNAc-β-1,6-GlcNAc-β (glycan 27), 4-P-GlcNAc-β-1,4-Man-β (glycan 28), Glc-α-1,2-Gal-α-1,3-Glc-α (glycan 29), Gal-β-1,4-GlcNAc-β-1,3-Gal-β-1,4-Glc-β-[LNnT] (glycan 37), GlcA-β-1,4-GlcNAc-α-1,4-GlcA-β (glycan 38), GalNAc-β-1,4-GlcNAc-β (glycan 41), Gal-α-1,2-Gal-α (glycan 48), Gal-β-1,4-(6S)GlcNAc-β (glycan 53), GalNAc-α-1,3-(Fuc-α-1,2)-Gal-β-[blood A antigen trisaccharide] (glycan 57), Gal-α-1,3-(Fuc-α-1,2)-Gal-β-[blood B antigen trisaccharide] (glycan 59), β-d-Rha-Sp (glycan 75), Glc-α-1,4-Glc-β (glycan 76), Glc-α-1,6-Glc-α-1,4-Glc-β (glycan 77), and SGP (sialylglycopeptide; glycan 100). Fig. 2B shows a representative array after binding by P. aeruginosa, including both negative and positive fluorescence controls. The quantification of binding of each of the 28 glycans is shown in Fig. 2C, with the glycans ordered by signal intensity. A summary of the glycan array binding data is provided in Table 1.

**TABLE 1:** Glycan-binding profile of P. aeruginosa PAO1^*a*^

Glycan no.	Glycan	MFI for:			
		Array 1	Array 2	Array 3	Avg
1	β-Glc-Sp	156.625	376.875	413.625	315.708
2	β-Gal-Sp	271.125	313.75	347.75	310.875
3	α-Man-Sp	133.5	160.625	157.375	150.5
4	α-Fuc-Sp	180.375	327.125	307	271.5
5	α-Rha-Sp	163.625	135.375	115.375	138.125
6	β-GlcNAc-Sp	505.125	441.875	429	458.666
7	β-GalNAc-Sp	512.875	345.125	360.875	406.291
8	Tobramycin	485.25	449.75	432.375	455.791
9	Gal-β-1,3-GlcNAc-β-Sp	250	225.375	203.75	226.375
10	Gal-α-1,3-Gal-β-1,3-GlcNAc-β-Sp	187.125	375.625	337.5	300.083
11	Neu5Ac-α-2,3-Gal-β-1,3-GlcNAc-β-Sp	157.75	186	140.875	161.541
12	Neu5Ac-α-2,6-Gal-β-1,3-GlcNAc-β-Sp	10.625	57.375	55.75	41.25
13	Neu5Gc-α-2,3-Gal-β-1,3-GlcNAc-β-Sp	20	34	21.5	25.166
14	Neu5Gc-α-2,6-Gal-β-1,3-GlcNAc-β-Sp	19.375	63.25	72.5	51.708
15	Neu5Gc-α-2,6-Gal-β-1,3-GlcNAc-β-Sp	9	105.5	38	50.833
16	Gal-β-1,4-Glc-β-Sp	17	104.75	64	61.916
17	Gal-α-1,3-Gal-β-1,4-Glc-β-Sp	26.25	89.875	63.875	60
18	Gal-α-1,4-Gal-β-1,4-Glc-β-Sp	62.125	128	68.125	86.083
19	GlcNAc-β-1,3-Gal-β-1,4-Glc-β-Sp	5	76.625	62.375	48
20	GalNAc-β-1,3-Gal-β-1,4-Glc-β-Sp	148.375	168.375	161	159.25
21	Neu5Ac-α-2,3-Gal-β-1,4-Glc-β-Sp	75	79	61.875	71.958
22	Neu5Ac-α-2,6-Gal-β-1,4-Glc-β-Sp	71.625	46.75	59.375	59.25
23	Neu5Gc-α-2,3-Gal-β-1,4-Glc-β-Sp	6.5	80.125	116.125	67.583
24	Neu5Ac-α-2,6-Gal-β-1,4-Glc-β-Sp	59.75	84.25	110.5	84.833
25	Gal-β-1,4-(Fuc-α-1,3)-Glc-β-Sp	149.25	121.25	177.875	149.458
26	GalNAc-β-1,3-Gal-α-1,4-Gal-β-1,4-Glc-β-Sp	49	48.5	22.75	40.083
27	GlcNAc-β-1,6-GlcNAc-β-Sp	73.625	114.375	117.875	101.958
28	4-P-GlcNAc-β-1,4-Man-β-Sp	108	202.5	138.625	149.708
29	Glc-α-1,2-Gal-α-1,3-Glc-α-Sp	143.5	94.25	88.25	108.667
30	Gal-β-1,3-GalNAc-α-Sp	36.75	35.875	38	36.875
31	Gal-β-1,4-GlcNAc-β-Sp	6.125	40.875	20.75	22.583
32	Gal-β-1,4-(Fuc-α-1,3)-GlcNAc-β-[Lewis X]–Sp	30.375	65.5	35.625	43.833
33	Neu5Ac-α-2,3-Gal-β-1,4-(Fuc-α-1,3)-GlcNAc-β-[sialyl Lewis X]-Sp	20.875	38.625	26.25	28.583
34	Neu5Ac-α-2,3-Gal-β-1,3-(Fuc-α-1,4)-GlcNAc-β-[sialyl Lewis A]-Sp	15	16.875	12.875	14.916
35	Neu5Gc-α-2,3-Gal-β-1,3-(Fuc-α-1,4)-GlcNAc-β- [sialyl Lewis A]-Sp	13.125	11.75	2.25	9.0416
36	Gal-α-1,4-Gal-β-1,3-GlcNAc-β-Sp	12.625	46.375	38	32.333
37	Gal-β-1,4-GlcNAc-β-1,3-Gal-β-1,4-Glc-β-[LNnT]-Sp	193.75	183.625	133.375	170.25
38	GlcA-β-1,4-GlcNAc-α-1,4-GlcA-β-Sp	122.25	115.25	89.625	109.041
39	GlcNAc-β-1,6-(Gal-β-1,3)-GalNAc-α-OSer-Sp4	34.625	44	8.25	28.958
40	Neu5Ac-α-2,3Gal-β-1,4-(6S)GlcNAc-β-Sp	28.25	36.25	55	39.833
41	GalNAc-β-1,4-GlcNAc-β-Sp2	110.625	103.875	86	100.166
42	Neu5Ac-α-2,8-Neu5Ac-α-2,3-Gal-β-1,4-Glc-β-Sp	65.625	56.125	27.75	49.833
43	Neu5Gc-α-2,8-Neu5Ac-α-2,3-Gal-β-1,4-Glc-β-Sp	16.25	37.5	50.875	34.875
44	GalNAc-α-1,3-(Fuc-α-1,2)-Gal-β-1,4-Glc-β-[blood A antigen tetrose]-Sp1	73.75	76.75	72.875	74.458
45	GlcNAc-β-1,2-Man-α-Sp	26.25	30	39.375	31.875
46	Neu5Ac-α-2,3-Gal-β-Sp1	10.25	35.125	27	24.125
47	Gal-β-1,3-GalNAc-β-1,3-Gal-β-Sp1	17.25	39.75	46.875	34.625
48	Glc-α-1,2-Gal-α-Sp	160.875	89.875	90.375	113.708
49	Gal-β-1,4-(Fuc-α-1,3)-GlcNAc-β-1,3-Gal-β-Sp1	5.75	60.875	52.5	39.708
50	Neu5Ac-α-2,3-Gal-β-1,4-(Fuc-α-1,3)-Glc-β-[3-sialyl-3-fucosyllactose/F-SL]-Sp1	28.625	74.875	57.75	53.75
51	GlcNAc-β-1,4-GlcNAc-β-Sp1	57	68.125	58.125	61.083
52	β-d-GlcA-Sp	23	132.625	107.25	87.625
53	Gal-β-1,4-(6S)GlcNAc-β-Sp	101	156.875	103.125	120.333
54	GlcNAc-α-1,3-(Glc-α-1,2-Glc-α-1,2)-Gal-α-1,3-Glc-α-Sp	57.375	31	47.625	45.3333
55	Gal-β-1,3-GalNAc-β-1,4-(Neu5Gc-α-2,3)-Gal-β-1,4-Glc-β-Sp1	6.875	98.125	95.875	66.958
56	Sisomicin sulfate	17.375	54.625	16.625	29.541
57	GalNAc-α-1,3-(Fuc-α-1,2)-Gal-β-[blood A antigen trisaccharide]-Sp1	163.75	154.625	123.75	147.375
58	Fuc-α-1,2-Gal-β-1,4-GlcNAc-β-[blood H antigen trisaccharide]-Sp1	68.125	94.875	62.625	75.208
59	Gal-α-1,3-(Fuc-α-1,2)-Gal-β-[blood B antigen trisaccharide]-Sp1	124.875	139.5	119.125	127.833
60	Fuc-α-1,2-Gal-β-1,3-GlcNAc-β-1,3-Gal-β-1,4-Glc-β-[LNFP I]-Sp1	52.5	84.25	104.375	80.375
61	Fuc-α-1,2-Gal-β-1,4-Glc-β- [blood H antigen trisaccharide]-Sp1	48.75	33.25	36.25	39.416
62	Gal-α-1,3-(Fuc-α-1,2)-Gal-β-1,4-Glc-β-[blood B antigen tetrasaccharide]-Sp1	18.75	25.75	27.375	23.958
63	(Fuc-α-1,2)-Gal-β-1,4-(Fuc-α-1,3)-GlcNAc-β-[Lewis Y]-Sp1	1.25	23.625	8.875	11.25
64	(Fuc-α-1,2)-Gal-β-1,3-(Fuc-α-1,4)-GlcNAc-β-[Lewis B]-Sp1	12.5	29.75	6.125	16.125
65	Gal-β-1,3-(Fuc-α-1,4)-GlcNAc-β-1,3-Gal-β-1,4-(Fuc-α-1,4)-Glc-β-[Lewis A]-Sp1	19.75	42.625	9	23.791
66	Gal-β-1,3-GalNAc-β-Sp1	14.625	18	30.125	20.916
67	Gal-β-1,3-(Neu5Ac-α-2,6)-GalNAc-β-Sp	3.25	4.5	14.75	7.5
68	Neu5Ac-α-2,6-Gal-β-1,3-GalNAc-β-Sp	42.125	27.625	39.125	36.291
69	Neu5Ac-α-2,6-Gal-β-1,3-(Neu5Ac-α-2,6)-GalNAc-β-Sp	53.875	56.625	45.5	52
70	Neu5Ac-α-2,3-Gal-β-1,3-(Neu5Ac-α-2,6)-GalNAc-β-Sp	70.25	93.25	21	61.5
71	Neu5Ac-α-2,6-(Neu5Ac-α-2,3)-Gal-β-1,3-GalNAc-β-Sp	4.625	5	1.25	3.625
72	GalNAc-β-1,4-(Neu5Ac-α-2,3)-Gal-β-1,4-Glc-β-[GM2]-Sp	4.5	37.25	75.625	39.125
73	GalNAc-β-1,4-(Neu5Ac-α-2,8-Neu5Ac-α-2,3)-Gal-β-1,4-Glc-β-[GD2]-Sp	31	49.75	17.625	32.791
74	Gal-α-1,4-Gal-β-1,4-GlcNAc-β-Sp1	92	27.625	87	68.875
75	β-d-Rha-Sp	254.875	294.375	263	270.75
76	Glc-α-1,4-Glc-β-Sp1	120.5	274.25	264.125	219.625
77	Glc-α-1,6-Glc-α-1,4-Glc-β-Sp1	157.125	132.125	75.75	121.666
78	Maltotriose-β-Sp1	52.5	100.5	26.875	59.958
79	Glc-α-1,6-Glc-α-1,6-Glc-β-Sp1	11	90.875	60.375	54.083
80	Maltotetraose-β-Sp1	89	71.5	63.5	74.666
81	GlcNAc-α-1,4-GlcA-β-1,4-GlcNAc-α1,4-GlcA-β-Sp	36.5	62.375	61.25	53.375
82	Maltohexaose-β-Sp1	8.5	44.875	24	25.791
83	Maltoheptaose-β-Sp1	28.625	47.5	28.875	35
84	Acarbose-β-Sp1	39.5	75	60.25	58.25
85	d-Pentamannuronic acid-β-Sp1	55	248.625	179.625	161.083
86	l-Pentaguluronic acid-β-Sp1	50.25	105.75	103.625	86.541
87	d-Cellose-β-Sp1	5	79.25	1.75	28.666
88	Gal-α-1,3-Gal-β-Sp1	26	153.5	118.5	99.333
89	β-1,4-xylotetrose-Sp1	42	122.25	94.125	86.125
90	Chitin-trisaccharide-Sp1	31.875	21.75	24.125	25.916
91	KDN-α-2,8-Neu5Ac-α-2,3-Gal-β-1,4-Glc-β-Sp	153.375	132	41	108.791
92	Neu5Ac-α-2,8-Neu5Gc-α-2,3-Gal-β-1,4- Glc-β-Sp	21.25	40.25	23.75	28.416
93	Neu5Ac-α-2,8-Neu5Ac-α-2,8-Neu5Ac-α-2,3-Gal-β-1,4-Glc-β-Sp3	15.875	68.125	40.125	41.375
94	Neu5Ac-α-2,8-Neu5Ac-α-2,6-Gal-β-1,4-Glc-Sp5	12.75	43.5	4.25	20.166
95	Gal-β-1,3-GalNAc-β-1,4-(Neu5Ac-α-2,3)-Gal-β-1,4-Glc-β-Sp1	21.5	105.5	54.125	60.375
96	Gentamicin sulfate	16.625	64.25	91	57.291
97	Kanamycin sulfate	30.5	66.75	142.75	80
98	Geneticin disulfate salt (G418)	12.25	36.5	37.75	28.833
99	Neomycin trisulfate	25.375	111.625	34.875	57.291
100	SGP	74.5	60.375	101.25	78.708

a Glycan identities and mean fluorescence intensities (MFIs) of GFP-expressing P. aeruginosa PAO1 cells binding to 100 immobilized glycans. MFIs for each array were calculated as the average of the four data points from each glycan, spotted in quadruplicate on each array. A composite average MFI from the data across the 3 arrays (*n* = 12 for each glycan moiety) is shown in the final column. Positive glycan partners for P. aeruginosa were identified by the MFI as being at least 100 RFUs over background and significantly greater than the negative control; 28 glycans met these criteria.

### Pseudomonas aeruginosa binds to monosaccharides and an aminoglycoside.

We further examined the binding results for each of the glycan classes spotted on the Glycan-100 array to identify the binding signatures. The glycans on the array can be divided into eight groups: (i) monosaccharides; (ii) aminoglycosides; (iii) disaccharides; (iv) blood groups, Lewis antigens, and fucosylated oligosaccharides; (v) globo-series glycolipids, milk oligosaccharides, and GAGs; (vi) natural oligosaccharides; (vii) gangliosides and sialylated oligosaccharides; and (viii) α-Gal and N-glycans. Notably, a majority of positive glycan signatures identified were to monosaccharides and disaccharides; 15 of the 28 significantly bound glycans fell into this category. This analysis also revealed that 14 out of the 28 glycans identified had at least one galactose moiety. Furthermore, 11 of the 28 glycans to which P. aeruginosa bound contained at least one GlcNAc moiety. We describe the binding of P. aeruginosa PAO1 cells to each specific glycan class below.

**FIG 3:**
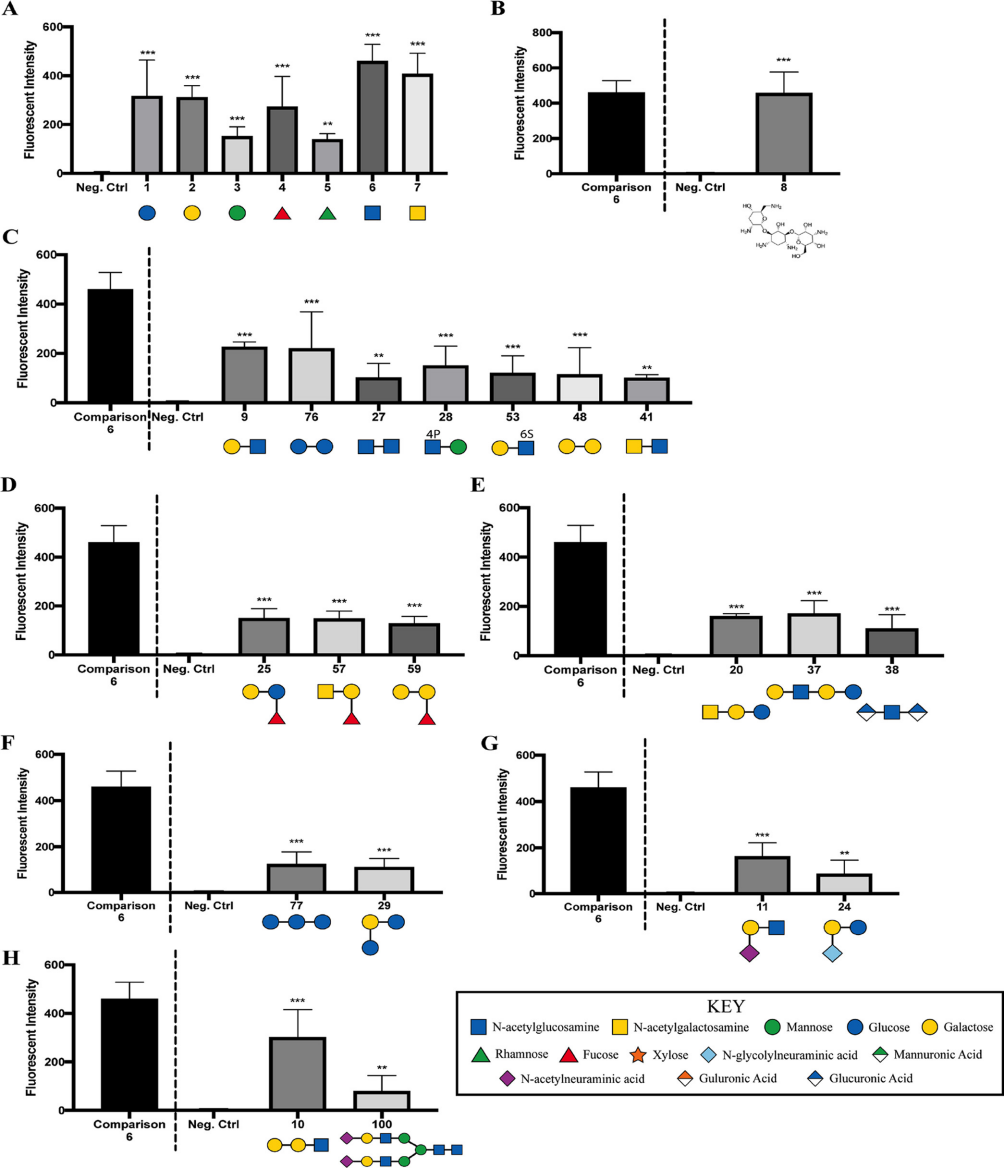
Glycan groups that exhibited significant binding by P. aeruginosa PAO1 cells, as measured using the Glycan-100 array: monosaccharides (A); an aminoglycoside (B); disaccharides (C); blood groups, Lewis antigens, and fucosylated oligosaccharides (D); globo-series glycolipids, milk oligosaccharides, and GAGs (E); natural oligosaccharides (F); gangliosides and sialylated oligosaccharides (G); and α-Gal and N-glycans (H). Data were normalized to binding of GFP-expressing P. aeruginosa PAO1 cells compared to the negative control; the dotted line separates glycan 6 (GlcNAc), which serves as a point of comparison with the other tested glycans. Data were analyzed using a one-way ANOVA with Dunnett’s *post hoc* analysis (A, C to H) or an unpaired *t* test with Welch’s correction (B) and are representative of three independent experiments, with each glycan spotted in quadruplicate (*n* = 12). ***, *P ≤ *0.0005; **, *P ≤ *0.005. The structures were modified with permission from the figures at https://www.raybiotech.com/.

The significant attachment of P. aeruginosa cells to monosaccharides included (i) glucose, (ii) galactose, (iii) mannose, (iv) fucose, (v) rhamnose, (vi) GlcNAc, and (vii) GalNAc (Fig. 3A). With the exception of glucose and rhamnose, these glycans are all components of N-linked glycan structures and are capable of competing for bacterial binding sites on phagocytes (Fig. 1). Furthermore, P. aeruginosa cells significantly bound tobramycin (Fig. 3B), an antibiotic used for the treatment of this microbe.

P. aeruginosa cells showed the most robust binding to Gal-β-1,3-GlcNAc (glycan 9) and Glc-α-1,4-Glc-β (glycan 76) in comparison to other disaccharides (Fig. 3C). Gal-β-1,3-GlcNAc has been described in complex and hybrid types of N-linked glycosylation (29). P. aeruginosa cells bound to the diamino-saccharide GlcNAc-β-1,6-GlcNAc (glycan 27) to a lesser extent than to the monoaminosaccharide GlcNAc (glycan 6) (Fig. 3A and C).

In addition to N-linked glycans, P. aeruginosa PAO1 cells also bound to selected blood groups, Lewis antigens, and fucosylated oligosaccharides (Fig. 3D); globo-series glycolipids, milk oligosaccharides, and GAGs (Fig. 3E); natural oligosaccharides (Fig. 3F); gangliosides and sialylated oligosaccharides (Fig. 3G); and α-Gal and N-glycans (Fig. 3H).

## DISCUSSION

P. aeruginosa cells can bind to glycans in a variety of contexts. Previous studies have shown that glycan moieties contribute to P. aeruginosa binding of airway epithelium (18, 19), and furthermore, bacterial surface adhesins can mediate binding to glycan ligands on host cells (32–35) and on other bacterial species (13, 15, 36). In addition, recent studies have revealed that the inhibition of N-linked glycan synthesis on host innate immune cells by tunicamycin decreased bacterial binding and, consequently, phagocytosis of P. aeruginosa and interleukin 1β (IL-1β) elicitation from the host cells (10). However, studies to date have interrogated a limited number of glycans that do not encompass all of the possible complexity of glycan structures (10, 11, 36). In this work, we combined cellular assays based on knowledge gained from previous studies with a screen to identify additional candidate glycans bound by P. aeruginosa.

Since P. aeruginosa cells preferentially bind to N-linked glycans (10), as illustrated in Fig. 1F, our initial efforts were focused on identification of the components of these glycans that may confer binding. Competition studies for bacterial binding and uptake revealed that specific monosaccharide components of N-linked glycans, including mannose and galactose, are effective at competing bacterial association with THP-1 monocytic cells, while in comparison other monosaccharides (including fucose), at equimolar concentrations, are much less effective. We note an important caveat: while we added the bacteria and glycans immediately before the phagocytic assays, we cannot rule out that these additions also impacted the activation/physiology of the THP-1 monocytic cells.

To further identify the breadth of glycans bound by P. aeruginosa, we performed a quantitative binding assay with GFP-labeled P. aeruginosa cells using the Glycan-100 array. Interestingly, we found that the majority of bound glycans have one or more galactose moieties, which aligns with the competition studies presented here (Fig. 1). We note that galactose was found in the following glycans identified in the array assay: Gal-β-1,4-(Fuc-α-1,3)-Glc (glycan 25), blood A antigen (glycan 57), blood B antigen (glycan 59), GalNAc-β-1,3-Gal-β-1,4-Glc (glycan 20), Gal-β-1,4-GlcNAc-β-1,3-Gal-β-1,4-Glc (glycan 37), Glc-α-1,2-Gal-α-1,3-Glc (glycan 29), Neu5Ac-α-2,3-Gal-β-1,3-GlcNAc (glycan 11), Neu5Gc-α-2,6-Gal-β-1,4-Glc (glycan 24), Gal-α-1,3-Gal-β-1,3-GlcNAc (glycan 10), and SGP (glycan 100). Similarly, mannose, identified in the competition assays, was also bound by P. aeruginosa in the array study, and GlcNac was shown to mediate robust binding by P. aeruginosa, despite only a modest effect on phagocytosis in the competition assays (Fig. 1). Overall, from the eight specified classes of glycans outlined above, monosaccharides, disaccharides, and an aminoglycoside were featured among the compounds bound by P. aeruginosa. Finally, we note that one limitation of this assay is that we cannot completely rule out some bacterial growth during the 3-h incubation from carryover of nutrients during the dilution of the culture 1:1,000 into the sample diluent buffer. Any such growth would simply enhance the relative binding signal, as growing bacteria that were not adhered to the array would be removed during the washing step.

How do our findings fit within the context of previous studies? A previous study by Ramphal and colleagues showed that P. aeruginosa binds two different Gal-GlcNAc disaccharides (25), consistent with our findings here (Fig. 2) (compounds 9 and 53). Similarly, this microbe binds to the GalNAcβ1-4Gal disaccharide (37), also consistent with our findings. Previous work also indicated that P. aeruginosa can bind *N*-acetylglucosamine and sialic acids (21), lipid-linked lactose and lactose-derivatives (24), and sialyl-Lewis X conjugates (22). We confirmed many of these findings here, supporting previous studies that showed the ability of P. aeruginosa to bind to specific moieties of glycolipids and mucins (20, 25) and serving as a validation of our studies. P. aeruginosa has also been shown to bind to chitin (27), but we did not address binding to this sugar polymer.

One particularly intriguing observation was that P. aeruginosa binds to tobramycin (glycan 8). Tobramycin is a broad-spectrum antibiotic that is administered via intravenous or intramuscular injection and is also utilized heavily by oral inhalation for treatment of CF-associated chronic infections (38). Tobramycin inhibits protein synthesis by binding to the 16S rRNA of the bacterial 30S ribosome, which leads to mistranslation and subsequent cell membrane damage (39). This study suggests a novel mechanism of interaction between P. aeruginosa and tobramycin, perhaps explaining tobramycin’s potency in killing this microbe (40). Additional studies will be needed to delineate how the observed binding may contribute to tobramycin’s antibiotic activity.

## MATERIALS AND METHODS

### Bacteria.

P. aeruginosa strain PAO1 carrying the GFP-expressing plasmid pSMC21 (41) was provided by D. Hogan and used here and in previous studies (4, 5, 42, 43). Bacteria were cultured overnight in LB at 37°C and subsequently subcultured to achieve log-phase growth for 2 h in LB. Bacterial growth was determined by optical density at 600 nm.

### Cell culture.

THP-1 human monocytic cells were provided by P. Guyre (Geisel School of Medicine at Dartmouth, Lebanon, NH). Using a modification of a previously published protocol (44), cells were cultured and maintained in RPMI 1640 medium (HyClone) supplemented with 10% fetal bovine serum (FBS; HyClone), 5% penicillin-streptomycin solution (Pen-Strep), 5% l-glutamine, and 1 mM sodium pyruvate until harvest. The phagocytic cells were not activated.

### Gentamicin protection assay.

Phagocytosis of live bacteria was performed and quantitated as previously described (4, 5, 9, 10). Briefly, overnight cultures of P. aeruginosa were washed, resuspended in serum-free Hanks balanced salt solution (HBSS; Corning), and added to the THP-1 cells at a multiplicity of infection (MOI) of 10. Where indicated, the phagocytic cells were treated with the specified glycan immediately before the competition studies, as previously described (10). The THP-1 cells were not activated prior to exposure to the bacteria or the glycans. After a 45-min coincubation of bacteria with the glycan or control, 100 μg/mL gentamicin was added to the assay for 20 min at 37°C. The cultures were washed and subsequently lysed in 500 μL 0.1% Triton X-100 solution in 1× phosphate-buffered saline (PBS). Lysates were plated on LB plates and incubated overnight at 37°C to determine viable bacterial counts. The next day, recovered CFU on the LB plates were enumerated and represented as the percentage of the mean of WT bacteria phagocytosed or the fold increase in phagocytosis, as indicated in the figure legends, to quantitatively compare the relative phagocytosis levels.

### Glycan-100 array analysis.

The commercially available Glycan-100 array (RayBiotech) was used to assess carbohydrate binding preferences to 100 described glycan structures by P. aeruginosa. To assess glycan binding by P. aeruginosa, live GFP-expressing P. aeruginosa PAO1 cells were subjected to glycan array analysis (*n* = 3). After growth overnight in LB medium, the GFP-expressing P. aeruginosa PAO1 cells were subcultured into fresh LB for ~2 h and then diluted 1:1,000 in the manufacturer’s sample diluent buffer for a final bacterial cell count of ~1 × 10^6^ CFU/mL. This suspension was applied to the array and incubated at room temperature for 3 h as per the manufacturer’s directions. The positive control was a fluorescent protein, and the negative control had nothing spotted on the array. Scanning and analysis of the slide were performed using the DNA Microarray Scanner (Agilent Technologies).

A list of the glycans on the Glycan-100 array is provided in Table 1. The glycan identities and mean fluorescence intensities (MFIs) of GFP-expressing P. aeruginosa PAO1 cells binding to the glycans were determined. The MFI for each glycan was calculated as the average of the four data points from each glycan (i.e., each glycan was spotted in quadruplicate on each array) from across the 3 arrays (thus, *n* = 12 for each glycan moiety). This average MFI is shown in the final column of Table 1. Positive glycan-binding partners for P. aeruginosa were set using an MFI at >100 relative fluorescence units (RFUs) over background and significantly greater than the negative control; 28 glycans met these criteria.

### Statistical analyses.

The means ± standard deviations (SD) derived from multiple independent experiments with technical replicates are shown for each graph. The sample sizes for each experiment are noted in the figure captions. As indicated in the captions, the unpaired Student’s *t* test with Welch’s correction or one-way analysis of variance (ANOVA) with Tukey’s *post hoc* analysis were performed using Prism version 7.02 to determine the statistical significance of the data. Statistical significance is represented in the figures by asterisks.

### Data availability.

The glycan array is listed in Table 1, with the P. aeruginosa glycan-binding profile.

## References

[1] Hauser AR, Jain M, Bar-Meir M, McColley SA. 2011. Clinical significance of microbial infection and adaptation in cystic fibrosis. Clin Microbiol Rev 24:29–70. doi:10.1128/CMR.00036-10. 21233507 PMC3021203

[2] Luzar MA, Thomassen MJ, Montie TC. 1985. Flagella and motility alterations in Pseudomonas aeruginosa strains from patients with cystic fibrosis: relationship to patient clinical condition. Infect Immun 50:577–582. doi:10.1128/iai.50.2.577-582.1985. 3932214 PMC261995

[3] Mahenthiralingam E, Campbell ME, Speert DP. 1994. Nonmotility and phagocytic resistance of Pseudomonas aeruginosa isolates from chronically colonized patients with cystic fibrosis. Infect Immun 62:596–605. doi:10.1128/iai.62.2.596-605.1994. 8300217 PMC186146

[4] Amiel E, Lovewell RR, O'Toole GA, Hogan DA, Berwin B. 2010. Pseudomonas aeruginosa evasion of phagocytosis is mediated by loss of swimming motility and is independent of flagellum expression. Infect Immun 78:2937–2945. doi:10.1128/IAI.00144-10. 20457788 PMC2897393

[5] Lovewell RR, Collins RM, Acker JL, O'Toole GA, Wargo MJ, Berwin B. 2011. Step-wise loss of bacterial flagellar torsion confers progressive phagocytic evasion. PLoS Pathog 7:e1002253. doi:10.1371/journal.ppat.1002253. 21949654 PMC3174259

[6] Andrews T, Sullivan KE. 2003. Infections in patients with inherited defects in phagocytic function. Clin Microbiol Rev 16:597–621. doi:10.1128/CMR.16.4.597-621.2003. 14557288 PMC207096

[7] Koh AY, Priebe GP, Ray C, Van Rooijen N, Pier GB. 2009. Inescapable need for neutrophils as mediators of cellular innate immunity to acute Pseudomonas aeruginosa pneumonia. Infect Immun 77:5300–5310. doi:10.1128/IAI.00501-09. 19805527 PMC2786465

[8] Akira S, Uematsu S, Takeuchi O. 2006. Pathogen recognition and innate immunity. Cell 124:783–801. doi:10.1016/j.cell.2006.02.015. 16497588

[9] Demirdjian S, Hopkins D, Sanchez H, Libre M, Gerber SA, Berwin B. 2018. Phosphatidylinositol-(3,4,5)-trisphosphate induces phagocytosis of nonmotile Pseudomonas aeruginosa. Infect Immun 86:e00215-18. doi:10.1128/IAI.00215-18. 29844235 PMC6056877

[10] Sanchez H, Hopkins D, Demirdjian S, Gutierrez C, O'Toole GA, Neelamegham S, Berwin B. 2021. Identification of cell-surface glycans that mediate motility-dependent binding and internalization of Pseudomonas aeruginosa by phagocytes. Mol Immunol 131:68–77. doi:10.1016/j.molimm.2020.12.012. 33358569 PMC8878251

[11] Varki A. 2017. Biological roles of glycans. Glycobiology 27:3–49. doi:10.1093/glycob/cww086. 27558841 PMC5884436

[12] van Kooyk Y, Rabinovich GA. 2008. Protein-glycan interactions in the control of innate and adaptive immune responses. Nat Immunol 9:593–601. doi:10.1038/ni.f.203. 18490910

[13] Sokurenko EV, Chesnokova V, Dykhuizen DE, Ofek I, Wu XR, Krogfelt KA, Struve C, Schembri MA, Hasty DL. 1998. Pathogenic adaptation of Escherichia coli by natural variation of the FimH adhesin. Proc Natl Acad Sci USA 95:8922–8926. doi:10.1073/pnas.95.15.8922. 9671780 PMC21178

[14] De Oliveira DM, Hartley-Tassell L, Everest-Dass A, Day CJ, Dabbs RA, Ve T, Kobe B, Nizet V, Packer NH, Walker MJ, Jennings MP, Sanderson-Smith ML. 2017. Blood group antigen recognition via the group A streptococcal M protein mediates host colonization. mBio 8:e02237-16. doi:10.1128/mBio.02237-16. 28119471 PMC5263248

[15] Rossez Y, Gosset P, Boneca IG, Magalhaes A, Ecobichon C, Reis CA, Cieniewski-Bernard C, Joncquel Chevalier Curt M, Leonard R, Maes E, Sperandio B, Slomianny C, Sansonetti PJ, Michalski JC, Robbe-Masselot C. 2014. The lacdiNAc-specific adhesin LabA mediates adhesion of Helicobacter pylori to human gastric mucosa. J Infect Dis 210:1286–1295. doi:10.1093/infdis/jiu239. 24755437

[16] Ofek I, Goldhar J, Keisari Y, Sharon N. 1995. Nonopsonic phagocytosis of microorganisms. Annu Rev Microbiol 49:239–276. doi:10.1146/annurev.mi.49.100195.001323. 8561460

[17] Byres E, Paton AW, Paton JC, Lofling JC, Smith DF, Wilce MC, Talbot UM, Chong DC, Yu H, Huang S, Chen X, Varki NM, Varki A, Rossjohn J, Beddoe T. 2008. Incorporation of a non-human glycan mediates human susceptibility to a bacterial toxin. Nature 456:648–652. doi:10.1038/nature07428. 18971931 PMC2723748

[18] Bucior I, Mostov K, Engel JN. 2010. Pseudomonas aeruginosa-mediated damage requires distinct receptors at the apical and basolateral surfaces of the polarized epithelium. Infect Immun 78:939–953. doi:10.1128/IAI.01215-09. 20008530 PMC2825949

[19] Bucior I, Pielage JF, Engel JN. 2012. Pseudomonas aeruginosa pili and flagella mediate distinct binding and signaling events at the apical and basolateral surface of airway epithelium. PLoS Pathog 8:e1002616. doi:10.1371/journal.ppat.1002616. 22496644 PMC3320588

[20] Ramphal R, Arora SK. 2001. Recognition of mucin components by Pseudomonas aeruginosa. Glycoconj J 18:709–713. doi:10.1023/a:1020823406840. 12386456

[21] Vishwanath S, Ramphal R. 1985. Tracheobronchial mucin receptor for Pseudomonas aeruginosa: predominance of amino sugars in binding sites. Infect Immun 48:331–335. doi:10.1128/iai.48.2.331-335.1985. 2985503 PMC261309

[22] Scharfman A, Arora SK, Delmotte P, Van Brussel E, Mazurier J, Ramphal R, Roussel P. 2001. Recognition of Lewis x derivatives present on mucins by flagellar components of Pseudomonas aeruginosa. Infect Immun 69:5243–5248. doi:10.1128/IAI.69.9.5243-5248.2001. 11500392 PMC98632

[23] Scharfman A, Degroote S, Beau J, Lamblin G, Roussel P, Mazurier J. 1999. Pseudomonas aeruginosa binds to neoglycoconjugates bearing mucin carbohydrate determinants and predominantly to sialyl-Lewis x conjugates. Glycobiology 9:757–764. doi:10.1093/glycob/9.8.757. 10406841

[24] Rosenstein IJ, Yuen CT, Stoll MS, Feizi T. 1992. Differences in the binding specificities of Pseudomonas aeruginosa M35 and Escherichia coli C600 for lipid-linked oligosaccharides with lactose-related core regions. Infect Immun 60:5078–5084. doi:10.1128/iai.60.12.5078-5084.1992. 1452340 PMC258280

[25] Ramphal R, Carnoy C, Fievre S, Michalski JC, Houdret N, Lamblin G, Strecker G, Roussel P. 1991. Pseudomonas aeruginosa recognizes carbohydrate chains containing type 1 (Gal beta 1-3GlcNAc) or type 2 (Gal beta 1-4GlcNAc) disaccharide units. Infect Immun 59:700–704. doi:10.1128/iai.59.2.700-704.1991. 1670932 PMC257814

[26] Ovchinnikova ES, Krom BP, Harapanahalli AK, Busscher HJ, van der Mei HC. 2013. Surface thermodynamic and adhesion force evaluation of the role of chitin-binding protein in the physical interaction between Pseudomonas aeruginosa and Candida albicans. Langmuir 29:4823–4829. doi:10.1021/la400554g. 23509956

[27] Folders J, Tommassen J, van Loon LC, Bitter W. 2000. Identification of a chitin-binding protein secreted by Pseudomonas aeruginosa. J Bacteriol 182:1257–1263. doi:10.1128/JB.182.5.1257-1263.2000. 10671445 PMC94410

[28] Delannoy CP, Rombouts Y, Groux-Degroote S, Holst S, Coddeville B, Harduin-Lepers A, Wuhrer M, Elass-Rochard E, Guerardel Y. 2017. Glycosylation changes triggered by the differentiation of monocytic THP-1 cell line into macrophages. J Proteome Res 16:156–169. doi:10.1021/acs.jproteome.6b00161. 27351377

[29] Stanley P, Taniguchi N, Aebi M. 2015. N-Glycans, p 99–111. *In* Varki A, Cummings RD, Esko JD, Stanley P, Hart GW, Aebi M, Darvill AG, Kinoshita T, Packer NH, Prestegard JH, Schnaar RL, Seeberger PH (ed), Essentials of glycobiology. Cold Spring Harbor Laboratory Press, Cold Spring Harbor, NY. doi:10.1101/glycobiology.3e.009. 27010055

[30] Demirdjian S, Hopkins D, Cumbal N, Lefort CT, Berwin B. 2020. Distinct contributions of CD18 integrins for binding and phagocytic internalization of Pseudomonas aeruginosa. Infect Immun 88:e00011-20. doi:10.1128/IAI.00011-20. 32041787 PMC7171243

[31] Zhang H, Brokman SM, Fang N, Pohl NL, Yeung ES. 2008. Linkage position and residue identification of disaccharides by tandem mass spectrometry and linear discriminant analysis. Rapid Commun Mass Spectrom 22:1579–1586. doi:10.1002/rcm.3550. 18433086

[32] Kuhaudomlarp S, Gillon E, Varrot A, Imberty A. 2020. LecA (PA-IL): a galactose-binding lectin from Pseudomonas aeruginosa. Methods Mol Biol 2132:257–266. doi:10.1007/978-1-0716-0430-4_25. 32306333

[33] Diggle SP, Stacey RE, Dodd C, Camara M, Williams P, Winzer K. 2006. The galactophilic lectin, LecA, contributes to biofilm development in Pseudomonas aeruginosa. Environ Microbiol 8:1095–1104. doi:10.1111/j.1462-2920.2006.001001.x. 16689730

[34] Chemani C, Imberty A, de Bentzmann S, Pierre M, Wimmerova M, Guery BP, Faure K. 2009. Role of LecA and LecB lectins in Pseudomonas aeruginosa-induced lung injury and effect of carbohydrate ligands. Infect Immun 77:2065–2075. doi:10.1128/IAI.01204-08. 19237519 PMC2681743

[35] Worstell NC, Singla A, Saenkham P, Galbadage T, Sule P, Lee D, Mohr A, Kwon JS, Cirillo JD, Wu HJ. 2018. Hetero-multivalency of Pseudomonas aeruginosa lectin LecA binding to model membranes. Sci Rep 8:8419. doi:10.1038/s41598-018-26643-7. 29849092 PMC5976636

[36] Poole J, Day CJ, von Itzstein M, Paton JC, Jennings MP. 2018. Glycointeractions in bacterial pathogenesis. Nat Rev Microbiol 16:440–452. doi:10.1038/s41579-018-0007-2. 29674747

[37] Krivan HC, Ginsburg V, Roberts DD. 1988. Pseudomonas aeruginosa and Pseudomonas cepacia isolated from cystic fibrosis patients bind specifically to gangliotetraosylceramide (asialo GM1) and gangliotriaosylceramide (asialo GM2). Arch Biochem Biophys 260:493–496. doi:10.1016/0003-9861(88)90473-0. 3124753

[38] Shawar RM, MacLeod DL, Garber RL, Burns JL, Stapp JR, Clausen CR, Tanaka SK. 1999. Activities of tobramycin and six other antibiotics against Pseudomonas aeruginosa isolates from patients with cystic fibrosis. Antimicrob Agents Chemother 43:2877–2880. doi:10.1128/AAC.43.12.2877. 10582875 PMC89580

[39] Kotra LP, Haddad J, Mobashery S. 2000. Aminoglycosides: perspectives on mechanisms of action and resistance and strategies to counter resistance. Antimicrob Agents Chemother 44:3249–3256. doi:10.1128/AAC.44.12.3249-3256.2000. 11083623 PMC90188

[40] Mah TF, Pitts B, Pellock B, Walker GC, Stewart PS, O'Toole GA. 2003. A genetic basis for Pseudomonas aeruginosa biofilm antibiotic resistance. Nature 426:306–310. doi:10.1038/nature02122. 14628055

[41] Davey ME, Caiazza NC, O'Toole GA. 2003. Rhamnolipid surfactant production affects biofilm architecture in Pseudomonas aeruginosa PAO1. J Bacteriol 185:1027–1036. doi:10.1128/JB.185.3.1027-1036.2003. 12533479 PMC142794

[42] Patankar YR, Lovewell RR, Poynter ME, Jyot J, Kazmierczak BI, Berwin B. 2013. Flagellar motility is a key determinant of the magnitude of the inflammasome response to Pseudomonas aeruginosa. Infect Immun 81:2043–2052. doi:10.1128/IAI.00054-13. 23529619 PMC3676033

[43] Toutain CM, Zegans ME, O'Toole GA. 2005. Evidence for two flagellar stators and their role in the motility of Pseudomonas aeruginosa. J Bacteriol 187:771–777. doi:10.1128/JB.187.2.771-777.2005. 15629949 PMC543560

[44] Inaba K, Inaba M, Deguchi M, Hagi K, Yasumizu R, Ikehara S, Muramatsu S, Steinman RM. 1993. Granulocytes, macrophages, and dendritic cells arise from a common major histocompatibility complex class II-negative progenitor in mouse bone marrow. Proc Natl Acad Sci USA 90:3038–3042. doi:10.1073/pnas.90.7.3038. 8464920 PMC46232

